# A systematic review of local field potential physiomarkers in Parkinson’s disease: from clinical correlations to adaptive deep brain stimulation algorithms

**DOI:** 10.1007/s00415-022-11388-1

**Published:** 2022-10-08

**Authors:** Bernadette C. M. van Wijk, Rob M. A. de Bie, Martijn Beudel

**Affiliations:** 1 Department of Human Movement Sciences, Vrije Universiteit Amsterdam, Van der Boechorststraat 9, 1081 BT Amsterdam, The Netherlands; 2 Department of Neurology, Amsterdam University Medical Centers, Amsterdam Neuroscience, University of Amsterdam, Amsterdam, The Netherlands

**Keywords:** Parkinson’s disease, Deep brain stimulation, Subthalamic nucleus, Electrophysiology, Beta oscillations

## Abstract

Deep brain stimulation (DBS) treatment has proven effective in suppressing symptoms of rigidity, bradykinesia, and tremor in Parkinson’s disease. Still, patients may suffer from disabling fluctuations in motor and non-motor symptom severity during the day. Conventional DBS treatment consists of continuous stimulation but can potentially be further optimised by adapting stimulation settings to the presence or absence of symptoms through closed-loop control. This critically relies on the use of ‘physiomarkers’ extracted from (neuro)physiological signals. Ideal physiomarkers for adaptive DBS (aDBS) are indicative of symptom severity, detectable in every patient, and technically suitable for implementation. In the last decades, much effort has been put into the detection of local field potential (LFP) physiomarkers and in their use in clinical practice. We conducted a research synthesis of the correlations that have been reported between LFP signal features and one or more specific PD motor symptoms. Features based on the spectral beta band (~ 13 to 30 Hz) explained ~ 17% of individual variability in bradykinesia and rigidity symptom severity. Limitations of beta band oscillations as physiomarker are discussed, and strategies for further improvement of aDBS are explored.

## Introduction

Parkinson’s disease (PD) is a neurodegenerative disease leading to a wide range of motor and non-motor symptoms. To date, neither a cure nor disease-modifying therapies are available. Dopaminergic medication may adequately suppress initial symptoms but typically become less effective as the disease progresses. Late-stage PD patients may be referred for stereotactic procedures such as deep brain stimulation (DBS). On average, DBS treatment significantly reduces motor symptoms as measured with the Unified Parkinson’s Disease Rating Scale (UPDRS) [1–4]. However, clinical outcomes are variable across individuals, and stimulation-induced side-effects such as dyskinesia, dysarthria, and neuro-psychiatric symptoms are common [5]. DBS efficacy may also vary during the day within individuals as a result of concurrent medication-intake or physiological fluctuations. These challenges call for an optimisation of stimulation settings adjusted to the individual patient and in a time-dependent way.

To achieve this, so-called physiomarkers related to the severity of certain (non-)motor symptoms or states can help to optimally titrate stimulation. For example, DBS could be switched on based on the detection of a physiomarker that signals the presence of tremor, and switched off when the physiomarker is no longer detected. This form of DBS is called “adaptive” (aDBS) or “closed-loop” DBS [6] and is currently already applied as clinical care in some countries [7]. aDBS potentially reduces side-effects due to overstimulation, saves battery power consumption, and also holds promise for implementing symptom-specific stimulation settings. The success of aDBS applications critically depends on the quality and predictive value of the used physiomarker. Non-invasive electrophysiological signals such as the EEG and ECG are relatively easy to measure but may not have a clear relation to symptom severity. EMG and accelerometry are capable of detecting tremor and dyskinesia symptoms [8–10] but are also prone to confounding signals from voluntary movements. In general, the following criteria can be applied to judge the clinical usefulness of a certain physiomarker:

**Table:** 

(1) Indicative	Is the physiomarker sufficiently linked to the severity of fluctuating symptoms?
(2) Individual	Is the physiomarker detectable in every patient and patient-specific if needed?
(3) Implementable	Is the physiomarker (technically) capable of automatically titrating stimulation?

A primary candidate for extracting suitable physiomarkers is the local field potential (LFP) signal that can be recorded from the DBS electrode contacts that are not used for stimulation. Modern sensing-enabling neurostimulators such as the Medtronic Percept™ PC [11] have demonstrated that it is technically feasible to integrate LFP recordings and stimulation within the same DBS device. This has the advantage that neural activity can be recorded directly from the DBS target structures that are thought to be implicated in the disease. LFP signal features might therefore have a direct (causal or associative) relation with clinical symptoms. In the last decades, much effort has been put into the discovery of LFP physiomarkers in PD and in their use in clinical practice. In this systematic review, we provide an overview of the markers that have been studied, show their pooled effect sizes, and discuss to what extent they are indicative, individual, and implementable for successful aDBS treatment.

## Literature search and research synthesis

**Fig. 1:**
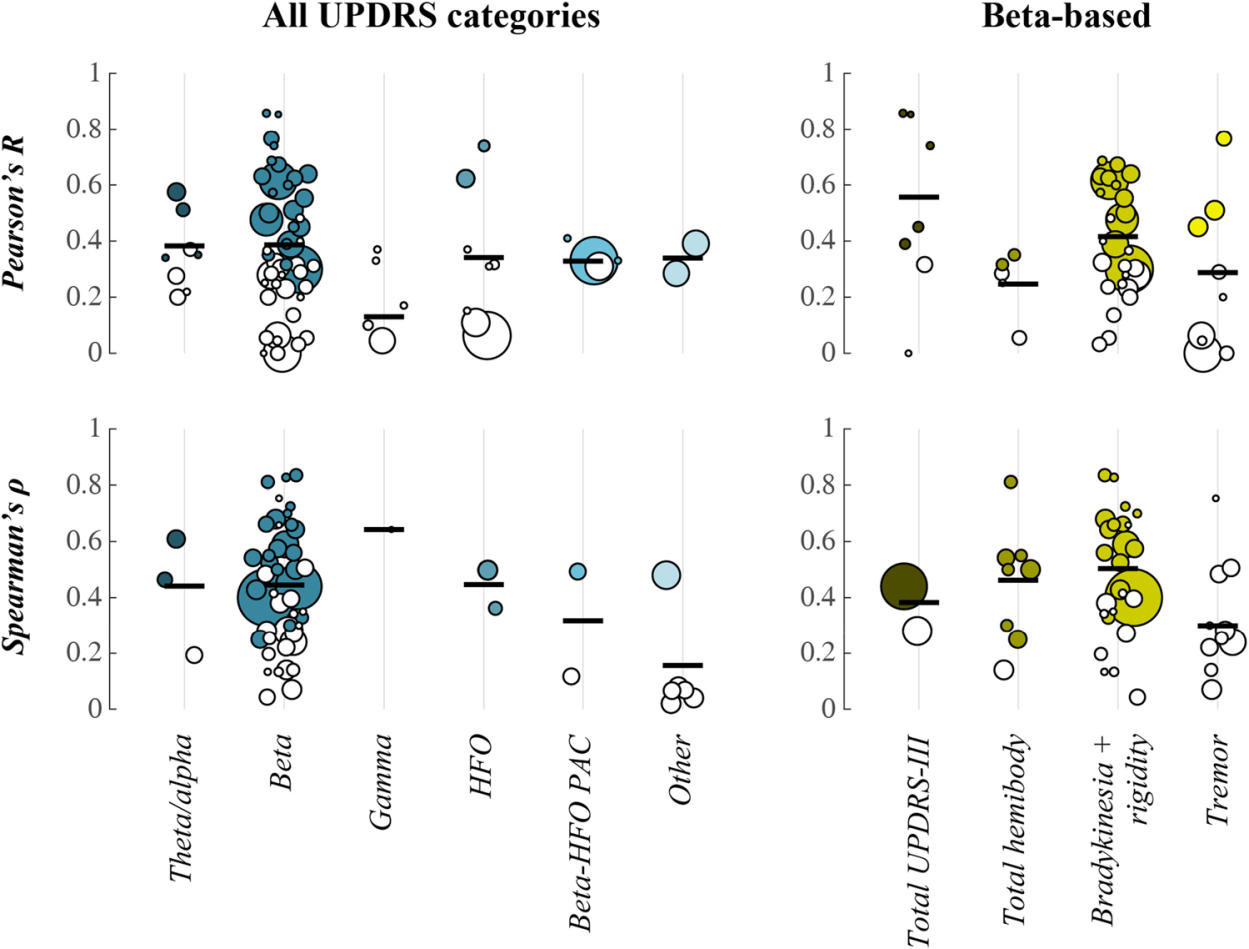
Distribution of correlation values and pooled effect sizes. Included data points are from Table 1 and 2. Filled circles represent correlation values that were deemed significant by the original study. Open circles represent non-significant correlation values. The size of circles reflects the number of hemispheres that were used in the correlation analysis. Black horizontal lines indicate the pooled effect size estimate across studies. Pearson’s *R* (top row) and Spearman’s *ρ* (bottom row) are visualised separately. All UPDRS categories include reported correlations for total UPDRS-III, total hemibody (bradykinesia + rigidity + tremor), hemibody bradykinesia + rigidity, and hemibody tremor items. These categories are visualised separately for beta-based LFP features on the right. Since the sign of the correlation was not necessarily comparable for different LFP measures, we opted for the use of absolute *R* and *ρ* values

The scientific literature was searched for studies that report outcomes of a correlation analysis between subthalamic nucleus (STN) LFP signal features and PD symptoms. The following search term was used in Web of Science: (LFP* OR "local field potential*") AND correlat* AND Parkinson*. This resulted in 270 abstracts that were scanned for relevance. Additional literature was collected through snowball sampling. Single subject cases and experimental studies in non-human species were excluded. Reported outcomes from Pearson (*R*) or Spearman (*ρ*) correlation analysis with UPDRS (sub-)scores were identified in the main text of the selected studies. These are presented separately for significant correlations in Table 1 and for non-significant correlations in Table 2. Considered categories are: total UPDRS-III score, total hemibody score (bradykinesia + rigidity + tremor items), hemibody bradykinesia + rigidity items, and hemibody tremor items. Pooled effect sizes were computed separately for *R* and *ρ* according to [12] and are displayed together with the distribution of data points in Fig. 1. A random-effects model was used to account for between-study heterogeneity with restricted maximum likelihood estimation of the heterogeneity variance.

**Table 1:** Inventory of significant correlations between STN-LFP signal features and UPDRS symptom severity

Study	*n* Patients/Hemispheres	LFP signal feature	UPDRS items	Medication state	Outcome^a^
**Beta-based**					
Doyle et al. (2005) [50]	14/14	Duration of movement-related spectral power decrease (13–35 Hz)	Total UPDRS-III	On, off	*R*^2^ = 0.20
	14/14	Amplitude of movement-related spectral power decrease (13–35 Hz)	Total UPDRS-III	On, off	*R*^2^ = 0.15
Kühn et al. (2006) [13]	9/17	Absolute spectral power within ± 2.5 Hz range around peak (8–35 Hz)	Total hemibody	Levodopa-induced changes	*ρ* = 0.81
	9/17	Absolute spectral power within ± 2.5 Hz range around peak (8–35 Hz)	Hemibody bradykinesia + rigidity	Levodopa-induced changes	*ρ* = 0.84
Ray et al. (2008) [51]	7/11	Absolute spectral peak power (8–35 Hz)	Hemibody bradykinesia + rigidity	Levodopa-induced changes	*ρ* = 0.70
Kühn et al. (2009) [14]	30/51	Absolute spectral power within ± 5.5 Hz range around peak (8–35 Hz)	Hemibody bradykinesia + rigidity	Levodopa-induced changes	*R*^2^ = 0.38
Chen et al. (2010) [26]	12/23	Lempel-Ziv complexity (13–35 Hz)	Total hemibody	Off	*ρ* = –0.54
	12/23	Lempel-Ziv complexity (13–35 Hz)	Hemibody bradykinesia + rigidity	Off	*ρ* = –0.53
Pogosyan et al. (2010) [23]	18/36	Phase coherence between unilateral contacts (13–35 Hz)	Hemibody bradykinesia + rigidity	Off	*R*^2^ = 0.15
López-Azcárate et al. (2010) [15]	14/26	Normalised spectral peak power (12–20 Hz)	Hemibody bradykinesia + rigidity	Off	*ρ* = 0.43
Özkurt et al. (2011) [16]	9/17	Absolute spectral peak power (8–35 Hz)	Hemibody bradykinesia + rigidity	On, off	*ρ* = 0.33
Little et al. (2012) [27]	18/36	Coefficient of variation spectral power over time (21–33 Hz)	Hemibody bradykinesia + rigidity	Off	*ρ* = –0.59
	10/17	Coefficient of variation spectral power over time (21–33 Hz)	Hemibody bradykinesia + rigidity	Levodopa-induced changes	*ρ* = –0.66
Hohlefeld et al. (2013) [52]	10/10	Imaginary part of coherency between unilateral contacts (10–30 Hz)^b^	Total UPDRS-III	Levodopa-induced changes	*R*^2^ = 0.55
Hohlefeld et al. (2014) [53]	8/8	Imaginary part of coherency between bilateral contacts (10–20 Hz)^b^	Total UPDRS-III	Off	*R*^2^ = 0.73
van Wijk et al. (2016) [18]	33/65	Normalised spectral power (13–20 Hz)	Hemibody bradykinesia + rigidity	On, off	*R*^2^ = 0.09
Neumann et al. (2016) [19]	63/63	Normalised spectral power (8–35 Hz)^b^	Total UPDRS-III	Off	*ρ* = 0.44
West et al. (2016) [21]	12/21	Absolute spectral power (13–20 Hz)	Hemibody bradykinesia + rigidity	Off	*R*^2^ = 0.39 *ρ* = 0.66
	12/22	Absolute spectral power (13–20 Hz)	Hemibody bradykinesia + rigidity	Levodopa-induced changes	*R*^2^ = 0.40 *ρ* = 0.56
	12/24	Coherence between unilateral contacts (13–20 Hz)	Hemibody bradykinesia + rigidity	Off	*R*^2^ = 0.41 *ρ* = 0.64
	12/24	Weighted phase lag index between unilateral contacts (13–20 Hz)	Hemibody bradykinesia + rigidity	Off	*R*^2^ = 0.31 *ρ* = 0.58
	12/12	Detrended fluctuation analysis of phase synchrony between bilateral contacts (13–20 Hz)^b^	Bradykinesia + rigidity	Off	*R*^2^ = 0.47 *ρ* = 0.73
	11/11	Detrended fluctuation analysis of phase synchrony between bilateral contacts (13–20 Hz)^b^	Bradykinesia + rigidity	Levodopa-induced changes	*R*^2^ = 0.33 *ρ* = 0.83
Beudel et al. (2017) [47]	39/78	Normalised spectral peak power (13–30 Hz)	Hemibody bradykinesia + rigidity	Off	*ρ* = 0.40
Neumann et al. (2017) [20]	12/24	Normalised spectral power within ± 3 Hz range around peak (13–35 Hz)	Total hemibody	On, off	*ρ* = 0.25
Tinkhauser et al. (2017) [24]	8/16	Percentage of beta bursts with long duration	Total hemibody	Off	*ρ* = 0.55
	8/16	Percentage of beta bursts with short duration	Total hemibody	Off	*ρ* = –0.30
	8/16	Median beta burst duration	Total hemibody	Levodopa-induced changes	*ρ* = 0.50
Tinkhauser et al. (2017) [25]	13/16	Percentage of beta bursts with long duration	Total hemibody	Off	*R*^2^ = 0.12
	13/16	Percentage of beta bursts with short duration	Total hemibody	Off	*R*^2^ = 0.10
Martin et al. (2018) [54]	13/26	Normalised spectral peak power (13–35 Hz)	Total hemibody	Off	*ρ* = 0.50
	13/26	Normalised spectral peak power (13–35 Hz)	Hemibody bradykinesia + rigidity	Off	*ρ* = 0.68
Özkurt et al. (2020) [17]	14/26	Nonlinearity of time series (13–30 Hz)	Hemibody tremor	Off	*R*^2^ = 0.20
	14/26	Normalised spectral power (13–30 Hz)	Hemibody bradykinesia + rigidity	Off	*R*^2^ = 0.25
	14/26	Normalised spectral power (13–30 Hz)	Hemibody tremor	Off	*R*^2^ = 0.26
Tamir et al. (2020) [22]	8/12	Normalised spectral power (13–30 Hz)	Hemibody bradykinesia + rigidity	Off	*R*^2^ = 0.36
Nie et al. (2021) [55]	10/10	Percentage of beta bursts with long duration^b^	Total UPDRS-III	Off	*ρ* = 0.74
	10/20	Percentage of beta bursts with long duration	Hemibody tremor	Off	*ρ* = 0.59
	10/20	Percentage of beta bursts with long duration	Hemibody rigidity^c^	Off	*ρ* = 0.45
Sure et al. (2021) [56]	24/44	Beta burst duration	Hemibody bradykinesia + rigidity	Off	*R*^2^ = 0.23
**Other**					
Pogosyan et al. (2010) [23]	18/36	Phase coherence between unilateral contacts (8–12 Hz)	Hemibody tremor	Off	*R*^2^ = 0.15
López-Azcárate et al. (2010) [15]	14/26	Normalised spectral peak power (250–350 Hz)	Hemibody bradykinesia + rigidity	Off	*ρ* = 0.50
	14/24	Movement-related changes in spectral peak power (250–350 Hz)	Hemibody bradykinesia + rigidity	Off	*R*^2^ = 0.39
	14/22	Phase-amplitude coupling (10–30 vs 200–400 Hz)	Hemibody bradykinesia + rigidity	Off	*ρ* = 0.49
Özkurt et al. (2011) [16]	9/18	Ratio of spectral power ± 10 Hz around slow (200–300 Hz) and fast peaks (300–400 Hz)	Hemibody bradykinesia + rigidity	On, off	*ρ* = 0.36
Giannicola et al. (2013) [57]	18/18	Normalised spectral power (2–7 Hz)	Total UPDRS-III	On	*R*^2^ = 0.26
Wang et al. (2014) [58]	10/15	Spectral peak power (160–470 Hz)	Hemibody bradykinesia + rigidity	Off	*R*^2^ = 0.55
van Wijk et al. (2016) [18]	33/65	Phase-amplitude coupling (13–20 vs 150–400 Hz)	Hemibody bradykinesia + rigidity	On, off	*R*^2^ = 0.11
West et al. (2016) [21]	12/24	Absolute spectral power (5–12 Hz)	Hemibody bradykinesia + rigidity	Off	*R*^2^ = 0.33 *ρ* = –0.61
Ozturk et al. (2020) [59]	9/9	Normalised spectral power (4–12 Hz)	Total hemibody	Levodopa-induced changes	*R*^2^ = 0.12
	9/9	Normalised spectral power (4–12 Hz)	Hemibody bradykinesia + rigidity	Levodopa-induced changes	*R*^2^ = 0.12
	9/9	Normalised phase-amplitude coupling (13–22 vs 200–300 Hz)	Total hemibody	Levodopa-induced changes	*R*^2^ = 0.11
	9/9	Normalised phase-amplitude coupling (13–22 vs 200–300 Hz)	Hemibody bradykinesia + rigidity	Levodopa-induced changes	*R*^2^ = 0.17
Weber et al. (2020) [60]	19/38	Differential entropy	Hemibody bradykinesia^c^	Off	*ρ* = 0.48
Belova et al. (2021) [61]	22/35	Movement-related change in 1/f spectral slope	Total UPDRS-III	Off	*R*^2^ = 0.08
Nie et al. (2021) [55]	10/20	Percentage of theta bursts with long duration	Hemibody tremor	Off	*ρ* = 0.46

^a^Outcomes are reported as explained variance (*R*^2^) computed from Pearson’s correlation coefficient or as Spearman’s rho (*ρ*). Values are rounded to two digits

^b^Total UPDRS-III and UPDRS scores for bilateral signal features (e.g. connectivity measures) were not lateralised. The number of included hemispheres is adjusted accordingly

^c^Correlations for bradykinesia and rigidity items are listed separately as average in case no combined bradykinesia + rigidity category was included in the original study

**Table 2:** Inventory of non-significant correlations between STN-LFP signal features and UPDRS symptom severity

Study	*n* Patients/Hemispheres	LFP signal feature	UPDRS items	Medication state	Outcome^a^
**Beta-based**					
Kühn et al. (2006) [13]	9/17	Absolute spectral power within ± 2.5 Hz range around peak (8–35 Hz)	Hemibody tremor	Levodopa-induced changes	*ρ* = 0.25 *p* = 0.54
Marceglia et al. (2006) [62]	21/21	Absolute spectral power (13–20 Hz)^b^	Total UPDRS-III	Off	*R*^2^ = 0.10 *p* = 0.14
Ray et al. (2008) [51]	5/9	Absolute spectral peak power (8–35 Hz)	Hemibody bradykinesia + rigidity	Off	*ρ* = –0.35 *p* = 0.15
	5/9	Absolute spectral peak power (8–35 Hz)	Hemibody tremor	Levodopa-induced changes	*ρ* = –0.30 *p* = 0.15
Kühn et al. (2009) [14]	30/51	Absolute spectral power within ± 5.5 Hz range around peak (8–35 Hz)	Hemibody tremor	Levodopa-induced changes	*R*^2^ = 0.00 *p* = 0.99
Chen et al. (2010) [26]	12/23	Lempel-Ziv complexity 13–35 Hz	Hemibody tremor	Off	*ρ* = –0.22 *p* = 0.31
	12/23	Absolute spectral power (13–35 Hz)	Hemibody tremor	Off	*ρ* = 0.48
	12/23	Normalised spectral power (13–35 Hz)	Hemibody tremor	Off	*ρ* = 0.51
Pogosyan et al. (2010) [23]	18/36	Phase coherence between unilateral contacts (13–35 Hz)	Hemibody tremor	Off	*R*^2^ = 0.00
López-Azcárate et al. (2010) [15]	14/26	Normalised spectral peak power (12–20 Hz)	Hemibody tremor	Off	
Little et al. (2012) [27]	18/36	Coefficient of variation spectral power over time (21–33 Hz)	Hemibody tremor	Off	*ρ* = –0.24 *p* = 0.16
	10/17	Coefficient of variation spectral power over time (21–33 Hz)	Hemibody tremor	Levodopa-induced changes	*ρ* = 0.14 *p* = 0.60
Hohlefeld et al. (2013) [52]	10/10	Imaginary part of coherency between unilateral contacts (10–30 Hz)^b^	Total UPDRS-III	Off	
	10/19	Imaginary part of coherency between unilateral contacts (10–30 Hz)^b^	Total UPDRS-III	On	
Hohlefeld et al. (2014) [53]	8/8	Coherence between bilateral contacts (10–20 Hz)^b^	Total UPDRS-III	Off	*R*^2^ = 0.00 *p* = 1.00
	8/8	Coherence between bilateral contacts (10–20 Hz)^b^	Total UPDRS-III	On	
	8/8	Coherence between bilateral contacts (10–20 Hz)^b^	Total UPDRS-III	Levodopa-induced changes	
	8/8	Imaginary part of coherency between bilateral contacts (10–20 Hz)^b^	Total UPDRS-III	On	
	8/8	Imaginary part of coherency between bilateral contacts (10–20 Hz)^b^	Total UPDRS-III	Levodopa-induced changes	
van Wijk et al. (2016) [18]	19/38	Normalised spectral power (13–20 Hz)	Hemibody bradykinesia + rigidity	Levodopa-induced changes	*R*^2^ = 0.08 *p* = 0.09
West et al. (2016) [21]	12/23	Coherence between unilateral contacts (13–20 Hz)	Hemibody bradykinesia + rigidity	Levodopa-induced changes	*R*^2^ = 0.09 *ρ* = 0.40 *p* = 0.06
	11/11	Coherence between bilateral contacts (13–20 Hz)^b^	Bradykinesia + rigidity	Off	*R*^2^ = 0.23 *ρ* = 0.42 *p* = 0.21
	12/12	Coherence between bilateral contacts (13–20 Hz)^b^	Bradykinesia + rigidity	Levodopa-induced changes	*R*^2^ = 0.06 *ρ* = 0.13 *p* = 0.68
	12/24	Weighted phase lag index between unilateral contacts (13–20 Hz)	Hemibody bradykinesia + rigidity	Levodopa-induced changes	*R*^2^ = 0.11 *ρ* = 0.27 *p* = 0.20
	11/11	Weighted phase lag index between bilateral contacts (13–20 Hz)^b^	Bradykinesia + rigidity	Off	*R*^2^ = 0.13 *ρ* = 0.34 *p* = 0.30
	9/9	Weighted phase lag index between bilateral contacts (13–20 Hz)^b^	Bradykinesia + rigidity	Levodopa-induced changes	*R*^2^ = 0.08 *ρ* = 0.13 *p* = 0.74
	12/21	Detrended fluctuation analysis of phase synchrony between unilateral contacts (13–20 Hz)	Hemibody bradykinesia + rigidity	Off	*R*^2^ = 0.04 *ρ* = 0.04 *p* = 0.85
	12/17	Detrended fluctuation analysis of phase synchrony between unilateral contacts (13–20 Hz)	Hemibody bradykinesia + rigidity	Levodopa-induced changes	*R*^2^ = 0.10 *ρ* = –0.20 *p* = 0.45
Beudel et al. (2017) [47]	39/39	Normalised spectral power (8–35 Hz)^b^	Total UPDRS-III	Off	*ρ* = 0.28 *p* = 0.07
	39/78	Normalised spectral (peak) power (8–35 Hz)	Hemibody tremor	Off	
Neumann et al. (2017) [20]	12/24	Normalised spectral power within ± 3 Hz range around peak (13–35 Hz)	Total hemibody	Levodopa-induced changes	
Martin et al. (2018) [54]	13/26	Normalised spectral peak power (13–35 Hz)	Hemibody tremor	Off	*ρ* = –0.07 *p* = 0.74
	13/26	Absolute spectral peak power (13–35 Hz)	Total hemibody	Off	*ρ* = –0.14
	13/26	Absolute spectral peak power (13–35 Hz)	Hemibody bradykinesia + rigidity	Off	*ρ* = 0.38
	13/26	Absolute spectral peak power (13–35 Hz)	Hemibody tremor	Off	*ρ* = 0.28
Ozturk et al. (2020) [59]	9/9	Normalised spectral power (13–22 Hz)	Total hemibody	Levodopa-induced changes	*R*^2^ = 0.06 *p* = 0.20
	9/9	Normalised spectral power (13–22 Hz)	Hemibody bradykinesia + rigidity	Levodopa-induced changes	*R*^2^ = 0.16 *p* = 0.06
	9/9	Normalised spectral power (13–22 Hz)	Hemibody tremor	Levodopa-induced changes	*R*^2^ = 0.04 *p* = 0.75
Özkurt et al. (2020) [17]	14/26	Nonlinearity of time series (13–30 Hz)	Hemibody bradykinesia + rigidity	Off	*R*^2^ = 0.05 *p* = 0.36
Tamir et al. (2020) [22]	8/12	Normalised spectral power (13–30 Hz)	Hemibody tremor	Off	*R*^2^ = 0.00 *p* = 0.88
Eisinger et al. (2020) [63]	15/19	Absolute spectral peak power (12–30 Hz)	Total hemibody	Off	*R*^2^ = 0.00 *p* = 0.83
	15/19	Absolute spectral peak power (12–30 Hz)	Hemibody bradykinesia + rigidity^c^	Off	*R*^2^ = 0.01
	15/19	Absolute spectral peak power (12–30 Hz)	Hemibody tremor	Off	*R*^2^ = 0.00 *p* = 0.91
	15/19	Amplitude of movement-related power decrease (12–30 Hz)	Total hemibody	Off	*R*^2^ = 0.08 *p* = 0.25
	15/19	Amplitude of movement-related power decrease (12–30 Hz)	Hemibody bradykinesia + rigidity^c^	Off	*R*^2^ = 0.02
	15/19	Amplitude of movement-related power decrease (12–30 Hz)	Hemibody tremor	Off	*R*^2^ = 0.08 *p* = 0.24
	15/19	Beta burst duration	Hemibody bradykinesia/rigidity	Off	*R*^2^ = 0.00
	15/19	Beta burst amplitude	Hemibody bradykinesia/rigidity	Off	*R*^2^ = 0.00
Telkes et al. (2020) [64]	7/8	Normalised spectral power (13–20 Hz)	Bradykinesia/rigidity	Off	*ρ* = 0.66 *p* = 0.09
	7/8	Normalised spectral power (13–20 Hz)	Tremor	Off	*ρ* = 0.75 *p* = 0.11
**Other**					
Kühn et al. (2006) [13]	?/7	Absolute spectral power within ± 2.5 Hz range around peak (60–90 Hz)	Total hemibody	Levodopa-induced changes	*ρ* = –0.64 *p* = 0.12
Marceglia et al. (2006) [62]	21/21	Absolute spectral power (8–12 Hz)^b^	Total UPDRS-III	Off	*R*^2^ = 0.04 *p* = 0.36
	13/13	Absolute spectral power (60–90 Hz)^b^	Total UPDRS-III	On	*R*^2^ = 0.01 *p* = 0.64
	13/13	Absolute spectral power (260–340 Hz)^b^	Total UPDRS-III	On	*R*^2^ = 0.10 *p* = 0.28
Chen et al. (2010) [26]	12/23	Lempel-Ziv complexity 0–12 Hz	Hemibody bradykinesia + rigidity	Off	*ρ* = 0.07 *p* = 0.76
	12/23	Lempel-Ziv complexity 0–12 Hz	Hemibody tremor	Off	*ρ* = 0.07 *p* = 0.76
López-Azcárate et al. (2010) [15]	14/22	Movement-related changes in phase-amplitude coupling (10–30 vs 200–400 Hz)	Hemibody bradykinesia + rigidity	Off	*ρ* = 0.18 *p* = 0.60
	14/26	Normalised spectral peak power (250–350 Hz)	Hemibody tremor	Off	
	14/26	Movement-related changes in spectral peak power (250–350 Hz)	Hemibody tremor	Off	
	14/26	Phase-amplitude coupling (10–30 vs 200–400 Hz)	Hemibody tremor	Off	
	14/26	Movement-related changes in phase-amplitude coupling (10–30 vs 200–400 Hz)	Hemibody tremor	Off	
Giannicola et al. (2013) [57]	18/18	Normalised spectral power (2–7 Hz)	Total UPDRS-III	Off	*R*^2^ = 0.13 *p* = 0.13
van Wijk et al. (2016) [18]	33/65	Normalised spectral power (150–400 Hz)	Hemibody bradykinesia + rigidity	On, off	*R*^2^ = 0.00 *p* = 0.56
	19/38	Normalised spectral power (150–400 Hz)	Hemibody bradykinesia + rigidity	Levodopa-induced changes	*R*^2^ = 0.01 *p* = 0.51
	19/38	Phase-amplitude coupling (13–20 vs 150–400 Hz)	Hemibody bradykinesia + rigidity	Levodopa-induced changes	*R*^2^ = 0.10 *p* = 0.06
West et al. (2016) [21]	12/22	Absolute spectral power (5–12 Hz)	Hemibody bradykinesia + rigidity	Levodopa-induced changes	*R*^2^ = 0.08 *ρ* = –0.19 *p* = 0.39
Martin et al. (2018) [54]	13/26	1/f slope	Total hemibody	Off	*ρ* = 0.04
	13/26	1/f slope	Hemibody bradykinesia + rigidity	Off	*ρ* = 0.02
	13/26	1/f slope	Hemibody tremor	Off	*ρ* = 0.08
Ozturk et al. (2020) [59]	9/9	Normalised spectral power (4–12 Hz)	Hemibody tremor	Levodopa-induced changes	*R*^2^ = 0.05 *p* = 0.08
	9/9	Normalised spectral power (70–90 Hz)	Total hemibody	Levodopa-induced changes	*R*^2^ = 0.03 *p* = 0.35
	9/9	Normalised spectral power (70–90 Hz)	Hemibody bradykinesia + rigidity	Levodopa-induced changes	*R*^2^ = 0.14 *p* = 0.12
	9/9	Normalised spectral power (70–90 Hz)	Hemibody tremor	Levodopa-induced changes	*R*^2^ = 0.11 *p* = 0.17
	9/9	Normalised spectral power (200–400 Hz)	Total hemibody	Levodopa-induced changes	
	9/9	Normalised spectral power (200–400 Hz)	Hemibody bradykinesia + rigidity	Levodopa-induced changes	
	9/9	Normalised spectral power (200–400 Hz)	Hemibody tremor	Levodopa-induced changes	
	9/9	Ratio of normalised spectral power between slow (200–300 Hz) and fast bands (300–400 Hz)	Total hemibody	Levodopa-induced changes	*R*^2^ = 0.10 *p* = 0.08
	9/9	Ratio of normalised spectral power between slow (200–300 Hz) and fast bands (300–400 Hz)	Hemibody bradykinesia + rigidity	Levodopa-induced changes	*R*^2^ = 0.14 *p* = 0.06
	9/9	Ratio of normalised spectral power between slow (200–300 Hz) and fast bands (300–400 Hz)	Hemibody tremor	Levodopa-induced changes	*R*^2^ = 0.02 *p* = 0.37
Weber et al. (2020) [60]	19/38	Differential entropy	Hemibody rigidity	Off	
	19/38	Differential entropy	Hemibody tremor	Off	
Belova et al. (2021) [61]	22/35	Amplitude of movement-related power increase (30–60 Hz)	Total UPDRS-III	Off	*R*^2^ = 0.00 *p* = 0.60

^a^Outcomes are reported as explained variance (*R*^2^) computed from Pearson’s correlation coefficient or as Spearman’s rho (*ρ*). Values are rounded to two digits. The *R*^*2*^, *ρ*, or *p* value is left blank in case no information was provided by the original study

^b^Total UPDRS-III and UPDRS scores for bilateral signal features (e.g. connectivity measures) were not lateralised. The number of included hemispheres is adjusted accordingly

^c^Correlations for bradykinesia and rigidity items are listed separately as average in case no combined bradykinesia + rigidity category was included in the original study

## Overview of LFP physiomarkers in PD

Out of the LFP signal features that have been linked to Parkinsonian symptom severity so far, the most frequently reported associations are between UPDRS-III (motor) scores of hemibody rigidity and bradykinesia and measures of contralateral STN beta (~ 13 to 30 Hz) oscillations. Higher spectral power values [13–22], a larger spatial extent over which beta oscillations can be detected [21, 23], longer bursts of beta oscillations in ongoing time series [24, 25], lower complexity of time series [26], and fewer fluctuations in spectral power over time [27] have all been linked to more impairment. Pooled together, their estimated correlation value with bradykinesia and rigidity symptoms equalled 0.416 for *R* (95%CI: [0.340 0.486]; *Q* = 27.44, *p* = 0.440; *τ* = 0.080, 95%CI: [0.000 0.208]; *I*^*2*^ = 1.6%, 95%CI: [0.0 42.9]) and 0.504 for *ρ* (95%CI: [0.416 0.582], *Q*  = 31.00, *p* = 0.189; *τ* = 0.107, 95%CI: [0.000 0.308]; *I*^2^ = 19.4%, 95%CI: [0.0 50.2])*.* This suggests that around 17% (*R*^*2*^) of individual variability in symptom severity can be explained by beta-based LFP signal features.

Other cardinal Parkinsonian symptoms have also been associated with spectral features of STN recordings. Strikingly, UPDRS tremor scores are typically not correlated with time-averaged spectral beta power, but a transient suppression of beta power may be observed during time periods in which strong tremor occurs [28, 29]. In addition, the presence of tremor has been linked to several other frequency bands including theta [30], low-gamma [31, 32], high-frequency oscillations (HFO) [33, 34], and the tremor frequency itself [35]. Dyskinesia has been linked to both theta and gamma activity [36, 37], and postural instability and gait problems with theta, alpha, (high-)beta activity, and HFO [34, 38–40]. Non-motor symptoms such as those related to impulse control [41–44] and depression [45, 46] have also shown to be reflected in LFP recordings.

Even for a single physiomarker such as spectral beta power, there is considerable variation amongst studies in the exact methodology used for linking it to symptom severity (see Table 1). Approaches differ in the use of absolute versus normalised power spectra, peak values versus a mean across a range of frequencies, the exact frequency range that is considered, and the medication state of the patient during which the recordings were obtained. These choices may or may not affect the statistical relation of recordings with clinical outcome measures. For example, averaging spectral power across the entire 8–35 Hz frequency range might be less sensitive for detecting a significant correlation with bradykinesia/rigidity scores compared to selecting power values at individual beta peak frequencies [47]. Despite several reports of significant correlations between measures of spectral beta power and UPDRS-III (sub-)scores, multiple studies found non-significant relations (Table 2). It is unclear whether these negative findings resulted from methodological choices, a lack of statistical power, or a true lack of correlation.

A suitable physiomarker for aDBS applications should be capable of differentiating between states of symptom severity within an individual or hemisphere. One way to investigate this is to look for correlations between levodopa-induced changes in LFP signal features and changes in clinical scores. In general, this has revealed similar correlation values between spectral beta power measures and bradykinesia and rigidity scores compared to on and/or off medication states alone. Another way is to look at fluctuations that naturally occur over time within an individual. The focus has again been mostly on beta oscillations, which have been demonstrated to occur in brief periods of high-amplitude bursts in the STN [25]. Importantly, movements that are triggered around the time of a beta burst are performed slower [48], hence underscoring the potential of this physiomarker for aDBS. The duration of these bursts seems crucial to consider. While the presence of long-duration bursts might signal symptoms of bradykinesia/rigidity, short-duration bursts are associated with good clinical scores [24, 25]. To date, most aDBS applications have used beta bursts with a minimum duration and amplitude as physiomarker for triggering stimulation, with performance comparable but not superior to continuous DBS [49].

## Current limitations of LFP physiomarkers in PD

Despite the progress in the field of discovering LFP physiomarkers for PD, there are still some important limitations. To start with, the low percentage of explained variability in outcome measures means that the relevant information contained in current physiomarkers is relatively small. Explanations for this relate to both physiomarker detection as well as appropriate quantification of symptom severity. Clinical ratings used for correlation analysis with physiomarkers are often rater-dependent and, for this reason, not objective. This is especially the case for bradykinesia items in UPDRS scores [65]. The scoring of these items is also nonlinear, meaning that a larger worsening of symptoms is needed to progress from a medium to high score than it is to progress from a low to medium score. For these reasons, the use of automated symptom assessments by, for example, automated video analyses [66] or smartwatch-derived signals [10] could help to improve the (Pearson) correlation between physiomarker and symptom severity.

Low correlation values may also result from inter-individual differences in LFP signal quality and (patho-)physiology. Suboptimal placement of DBS electrodes, electrode impedance [57], presence of cardiac or movement artefacts [67], and hardware failures may affect physiomarker detection. Even if electrode contacts are placed correctly in the STN target, an LFP beta peak is not always evident in every patient. Multiple studies report that a clear spectral beta peak was not discernable in ~ 5 to 15% of recordings [13, 14, 16, 24, 51, 53, 68] and a clear relation between peak movement velocity and beta burst amplitude was observable in 9 out of 12 patients in the study by [48]. These findings show similarities with other neurological disorders such as epilepsy in which, in some instances, no EEG abnormalities are seen [69]. Conversely, spectral beta peaks and beta-HFO phase-amplitude coupling have also been observed in LFP recordings from dystonia patients [68], hence questioning the specificity of these markers for PD. It is conceivable that the absence of certain physiomarkers in individual patients is related to deviant symptom manifestation and/or underlying neurobiological factors. Different pathophysiological mechanisms may lead to a very similar presentation of motor impairment.

Arguably the most suitable physiomarkers for aDBS are the ones that directly reflect the underlying neurobiological cause of the symptom that is considered. These might however be difficult to identify in LFP recordings, as the signal originates from the summed electrical activity of large populations of neurons and is mainly sensitive to only the synaptic input neurons receive. Although some evidence exists that STN stimulation at 20 Hz can slow movements in humans [70–72], in experimental animal models of progressive PD, symptom onset can precede the emergence of an LFP spectral beta peak [73, 74], suggesting that STN and GPi beta oscillations are not causally involved. As a minimum, beta oscillations alone seem not sufficient to explain the full spectrum of Parkinsonian symptoms. The role of other frequency bands or the interaction between frequency bands could still be further explored. With further development of hardware and neurophysiological understanding, it might be that additional physiomarkers can be identified that are closer to true neurobiological causes.

## Combinations of physiomarkers in PD

Several strategies are being developed to overcome the limitations of current LFP physiomarkers for aDBS. One promising avenue is the simultaneous use of multiple signal features to monitor different symptoms in parallel. In theory, monitoring of the tremor frequency range [75] could be combined with the monitoring of beta (bradykinesia/rigidity) and gamma oscillations (dyskinesia) [76] to control stimulation. In this way, the amplitude of beta oscillations might act as a trigger for switching on or off the stimulation, while the stimulation amplitude can be controlled based on gamma band power. Alternatively, single physiomarkers could be based on multiple signal features. Multiple regression models that include information from different frequency bands may significantly increase the correlation with UPDRS symptoms but can also reveal that individual predictors are merely collinear [16, 17, 21, 55]. Shah et al. [77] recently demonstrated that a weighted combination of spectral power in different frequency bands improved prediction performance of best stimulation contact compared to a single LFP feature. In principle the weights could be optimised per hemisphere to find a combination of LFP features that works best for aDBS treatment in an individual patient.

Next to the use of multiple characteristics of a single signal, it could be advantageous to combine physiomarkers from different recording techniques. ECoG recordings from the motor cortex could be informative for decoding the patient’s voluntary movements or to obtain additional information about the severity of symptoms [78]. In fact, the first (animal) study on aDBS in PD [79] used cortical physiomarkers to apply subcortical aDBS. A fully implanted ECoG-based aDBS system has already found its clinical application in a patient with cervical dystonia where the detection of a motor cortical theta burst triggers STN stimulation [80]. An additional benefit of further developing this approach for PD would be the smaller influence of stimulation artefacts that might impede the detection of physiomarkers when stimulation is switched on. In a laboratory setting, accelerometers and EMG have been successfully used for adaptive control of essential tremor by triggering stimulation in ventrolateral thalamus based on the phase or amplitude of measured upper limb tremor [81, 82]. Physiomarkers from wearable technology might however be technically more difficult to embed into the implanted pulse generator for care outside the clinic.

## Translating physiomarkers to closed-loop DBS treatment

The first proof-of-principle for aDBS was obtained in externalised patients in the immediate post-operative phase with the amplitude of beta oscillations as physiomarker for bradykinesia and rigidity symptoms [83]. Several subsequent steps have led towards its application in a ‘care as usual’ setting. The potential of beta-band aDBS was demonstrated to further include the control of speech problems [84], freezing of gait [85], and dyskinesia [86], and was tested in chronic [87, 88] and at home settings [89]. With the dawn of commercially available implantable DBS devices that are capable of chronic LFP recordings and adaptive programming [7, 90], the actual clinical merit of this form of stimulation can now be trialled on a large scale.

Choosing the right physiomarker(s) for aDBS can be challenging. A causal relation between the physiomarker and clinical symptoms may not be essential for successful applications, but correlation coefficients should be sufficiently high and generalisable to recordings taken from other moments in time or individuals. A fairly large number of studies have now replicated the association between beta-band-based LFP signal features and bradykinesia/rigidity symptoms. In addition, some evidence exists that the correlation with beta power remains consistent over a time period of several months [20]. The reproducibility of other less-well studied LFP signal features remains to be established. So far, most aDBS studies have dealt with the continuous scale of beta oscillation amplitude by setting a fixed threshold for the detection of symptom occurrence. This, however, leads to the additional challenge of choosing the right threshold for an individual patient and time period. Eventually, automatic classification algorithms based on single or multiple signal features [91–93] might lead to more successful applications. Such algorithms could possibly be augmented with the detection of physiological states such as activity level or active movement preparation [78, 94] to make stimulation settings context specific.

Another critical factor for the success of aDBS treatment is the signal quality of the used physiomarker. Most correlations reported in Table 1 were obtained after computing spectral power from a recording of several minutes. For real-life applications, the physiomarker should be detectable in recordings of a few seconds to optimally benefit from the dynamic nature of closed-loop control. At present, LFP recordings from sensing-enabled DBS devices are prone to cardiac, stimulation, and movement-related artefacts [95]. The true impact of these artefacts on aDBS applications has not yet been established. The key question is to what extent the artefacts lead to erroneous loops of stimulation instead of closed loops based on pathological brain activity. One important trade-off in this regard is the capability of the DBS device to perform complex operations on the LFP signals versus processing speed and battery consumption. Since more and more pulse generators are becoming rechargeable and can make use of processing capacities outside the body, these limitations might be overcome in the future.

## Conclusion

In the last decades various studies have reported clinical correlations between electrophysiological activity in the STN and symptomatology of PD. Out of this work, beta oscillations have surfaced as the ‘physiomarker’ with most potential as a control parameter for adaptive DBS treatment. However, current applications would likely need to improve on all three criteria of clinical usefulness (indicative, individual, implementable) in order to progress from a performance that is similar to continuous stimulation [49]. Even though sensing-enabled DBS devices are now commercially available, in the absence of a clear LFP-based programming strategy their current use is restricted to academic DBS centres [96]. The wider clinical applicability would benefit from closed-loop algorithms that can automatically detect relevant physiomarkers for titrating stimulation with minimum intervention from clinical staff. Fortunately, developments in research and hardware technology are moving fast. The first clinical trials on aDBS are currently being conducted and will likely shape the future application of this treatment.

## References

[1] Krack P, Batir A, Van Blercom N (2003). Five-year follow-up of bilateral stimulation of the subthalamic nucleus in advanced Parkinson’s disease. N Engl J Med.

[2] Rodriguez-Oroz MC, Obeso JA, Lang AE (2005). Bilateral deep brain stimulation in Parkinson’s disease: A multicentre study with 4 years follow-up. Brain.

[3] Deuschl G, Schade-Brittinger C, Krack P (2006). A randomized trial of deep-brain stimulation for Parkinson’s disease. N Engl J Med.

[4] Weaver FM, Follett K, Stern M (2009). Bilateral deep brain stimulation vs best medical therapy for patients with advanced Parkinson disease: a randomized controlled trial. JAMA.

[5] Benabid AL, Chabardes S, Mitrofanis J, Pollak P (2009). Deep brain stimulation of the subthalamic nucleus for the treatment of Parkinson’s disease. Lancet Neurol.

[6] Beudel M, Brown P (2016). Adaptive deep brain stimulation in Parkinson’s disease. Park Relat Disord.

[7] Nakajima A, Shimo Y, Fuse A (2021). Case report: chronic adaptive deep brain stimulation personalizing therapy based on Parkinsonian state. Front Hum Neurosci.

[8] Rabelo AG, Neves LP, Paixão APS (2017). Objective assessment of bradykinesia estimated from the wrist extension in older adults and patients with Parkinson’s disease. Ann Biomed Eng.

[9] Martinez-Manzanera O, Roosma E, Beudel M (2016). A method for automatic and objective scoring of bradykinesia using orientation sensors and classification algorithms. IEEE Trans Biomed Eng.

[10] Powers R, Etezadi-Amoli M, Arnold EM (2021). Smartwatch inertial sensors continuously monitor real-world motor fluctuations in Parkinson’s disease. Sci Transl Med.

[11] Jimenez-Shahed J (2021). Device profile of the percept PC deep brain stimulation system for the treatment of Parkinson’s disease and related disorders. Expert Rev Med Devices.

[12] Harrer M, Cuijpers P, Furukawa TA, Ebert DD (2021). Doing meta-analysis with R: a hands-on guide.

[13] Kühn AA, Kupsch A, Schneider G-H, Brown P (2006). Reduction in subthalamic 8–35 Hz oscillatory activity correlates with clinical improvement in Parkinson’s disease. Eur J Neurosci.

[14] Kühn AA, Tsui A, Aziz T (2009). Pathological synchronisation in the subthalamic nucleus of patients with Parkinson’s disease relates to both bradykinesia and rigidity. Exp Neurol.

[15] López-Azcárate J, Tainta M, Rodríguez-Oroz MC (2010). Coupling between beta and high-frequency activity in the human subthalamic nucleus may be a pathophysiological mechanism in Parkinson’s disease. J Neurosci.

[16] Özkurt TE, Butz M, Homburger M (2011). High frequency oscillations in the subthalamic nucleus: A neurophysiological marker of the motor state in Parkinson’s disease. Exp Neurol.

[17] Özkurt TE, Akram H, Zrinzo L (2020). Identification of nonlinear features in cortical and subcortical signals of Parkinson’s Disease patients via a novel efficient measure. Neuroimage.

[18] van Wijk BCM, Beudel M, Jha A (2016). Subthalamic nucleus phase-amplitude coupling correlates with motor impairment in Parkinson’s disease. Clin Neurophysiol.

[19] Neumann W-J, Degen K, Schneider G-H (2016). Subthalamic synchronized oscillatory activity correlates with motor impairment in patients with Parkinson’s disease. Mov Disord.

[20] Neumann WJ, Staub-Bartelt F, Horn A (2017). Long term correlation of subthalamic beta band activity with motor impairment in patients with Parkinson’s disease. Clin Neurophysiol.

[21] West T, Farmer S, Berthouze L (2016). The Parkinsonian subthalamic network: Measures of power, linear, and non-linear synchronization and their relationship to L-DOPA treatment and OFF state motor severity. Front Hum Neurosci.

[22] Tamir I, Wang D, Chen W (2020). Eight cylindrical contact lead recordings in the subthalamic region localize beta oscillations source to the dorsal STN. Neurobiol Dis.

[23] Pogosyan A, Yoshida F, Chen CC (2010). Parkinsonian impairment correlates with spatially extensive subthalamic oscillatory synchronization. Neuroscience.

[24] Tinkhauser G, Pogosyan A, Tan H (2017). Beta burst dynamics in Parkinson’s disease OFF and ON dopaminergic medication. Brain.

[25] Tinkhauser G, Pogosyan A, Little S (2017). The modulatory effect of adaptive deep brain stimulation on beta bursts in Parkinson’s disease. Brain.

[26] Chen CC, Hsu YT, Chan HL (2010). Complexity of subthalamic 13–35 Hz oscillatory activity directly correlates with clinical impairment in patients with Parkinson’s disease. Exp Neurol.

[27] Little S, Pogosyan A, Kühn AA, Brown P (2012). Beta band stability over time correlates with Parkinsonian rigidity and bradykinesia. Exp Neurol.

[28] Qasim SE, de Hemptinne C, Swann NC (2016). Electrocorticography reveals beta desynchronization in the basal ganglia-cortical loop during rest tremor in Parkinson’s disease. Neurobiol Dis.

[29] Shreve LA, Velisar A, Malekmohammadi M (2017). Subthalamic oscillations and phase amplitude coupling are greater in the more affected hemisphere in Parkinson’s disease. Clin Neurophysiol.

[30] Contarino MF, Bour LJ, Bot M (2012). Tremor-specific neuronal oscillation pattern in dorsal subthalamic nucleus of parkinsonian patients. Brain Stimul.

[31] Weinberger M, Hutchison WD, Lozano AM (2009). Increased gamma oscillatory activity in the subthalamic nucleus during tremor in Parkinson’s disease patients. J Neurophysiol.

[32] Beudel M, Little S, Pogosyan A (2015). Tremor reduction by deep brain stimulation is associated with gamma power suppression in Parkinson’s disease. Neuromodulation.

[33] Hirschmann J, Butz M, Hartmann CJ (2016). Parkinsonian rest tremor is associated with modulations of subthalamic high-frequency oscillations. Mov Disord.

[34] Telkes I, Viswanathan A, Jimenez-Shahed J (2018). Local field potentials of subthalamic nucleus contain electrophysiological footprints of motor subtypes of Parkinson’s disease. Proc Natl Acad Sci USA.

[35] Reck C, Florin E, Wojtecki L (2009). Characterisation of tremor-associated local field potentials in the subthalamic nucleus in Parkinson’s disease. Eur J Neurosci.

[36] Alonso-Frech F, Zamarbide I, Alegre M (2006). Slow oscillatory activity and levodopa-induced dyskinesias in Parkinson’s disease. Brain.

[37] Alegre M, López-Azcárate J, Alonso-Frech F (2012). Subthalamic activity during diphasic dyskinesias in Parkinson’s disease. Mov Disord.

[38] Syrkin-Nikolau J, Koop MM, Prieto T (2017). Subthalamic neural entropy is a feature of freezing of gait in freely moving people with Parkinson’s disease. Neurobiol Dis.

[39] Chen CC, Yeh CH, Chan HL (2019). Subthalamic nucleus oscillations correlate with vulnerability to freezing of gait in patients with Parkinson’s disease. Neurobiol Dis.

[40] Godinho F, Fim Neto A, Bianqueti BL (2021). Spectral characteristics of subthalamic nucleus local field potentials in Parkinson’s disease: phenotype and movement matter. Eur J Neurosci.

[41] Rodriguez-Oroz MC, López-Azcárate J, Garcia-Garcia D (2011). Involvement of the subthalamic nucleus in impulse control disorders associated with Parkinson’s disease. Brain.

[42] Rosa M, Fumagalli M, Giannicola G (2013). Pathological gambling in Parkinson’s disease: Subthalamic oscillations during economics decisions. Mov Disord.

[43] Mazzoni A, Rosa M, Carpaneto J (2018). Subthalamic neural activity patterns anticipate economic risk decisions in gambling. eNeuro.

[44] Ricciardi L, Fischer P, Mostofi A (2021). Neurophysiological correlates of trait impulsivity in Parkinson’s disease. Mov Disord.

[45] Huebl J, Schoenecker T, Siegert S (2011). Modulation of subthalamic alpha activity to emotional stimuli correlates with depressive symptoms in Parkinson’s disease1. Mov Disord.

[46] Sun Y, Wang Z, Hu K (2021). α and θ oscillations in the subthalamic nucleus are potential biomarkers for Parkinson’s disease with depressive symptoms. Park Relat Disord.

[47] Beudel M, Oswal A, Jha A (2017). Oscillatory beta power correlates with akinesia-rigidity in the parkinsonian subthalamic nucleus. Mov Disord.

[48] Torrecillos F, Tinkhauser G, Fischer P (2018). Modulation of beta bursts in the subthalamic nucleus predicts motor performance. J Neurosci.

[49] Little S, Brown P (2020). Debugging adaptive deep brain stimulation for Parkinson’s disease. Mov Disord.

[50] Doyle LMF, Kühn AA, Hariz M (2005). Levodopa-induced modulation of subthalamic beta oscillations during self-paced movements in patients with Parkinson’s disease. Eur J Neurosci.

[51] Ray NJ, Jenkinson N, Wang S (2008). Local field potential beta activity in the subthalamic nucleus of patients with Parkinson’s disease is associated with improvements in bradykinesia after dopamine and deep brain stimulation. Exp Neurol.

[52] Hohlefeld FU, Huchzermeyer C, Huebl J (2013). Functional and effective connectivity in subthalamic local field potential recordings of patients with parkinson’s disease. Neuroscience.

[53] Hohlefeld FU, Huchzermeyer C, Huebl J (2014). Interhemispheric functional interactions between the subthalamic nuclei of patients with Parkinson’s disease. Eur J Neurosci.

[54] Martin S, Iturrate I, Chavarriaga R (2018). Differential contributions of subthalamic beta rhythms and 1/f broadband activity to motor symptoms in Parkinson’s disease. NPJ Park Dis.

[55] Nie Y, Luo H, Li X (2021). Subthalamic dynamic neural states correlate with motor symptoms in Parkinson’s Disease. Clin Neurophysiol.

[56] Sure M, Vesper J, Schnitzler A, Florin E (2021). Dopaminergic modulation of spectral and spatial characteristics of parkinsonian subthalamic nucleus beta bursts. Front Neurosci.

[57] Giannicola G, Rosa M, Marceglia S (2013). The effects of levodopa and deep brain stimulation on subthalamic local field low-frequency oscillations in parkinson’s disease. Neurosignals.

[58] Wang J, Hirschmann J, Elben S (2014). High-frequency oscillations in Parkinson’s disease: Spatial distribution and clinical relevance. Mov Disord.

[59] Ozturk M, Abosch A, Francis D (2020). Distinct subthalamic coupling in the ON state describes motor performance in Parkinson’s disease. Mov Disord.

[60] Weber I, Florin E, von Papen M (2020). Characterization of information processing in the subthalamic area of Parkinson’s patients. Neuroimage.

[61] Belova EM, Semenova U, Gamaleya AA (2021). Voluntary movements cause beta oscillations increase and broadband slope decrease in the subthalamic nucleus of parkinsonian patients. Eur J Neurosci.

[62] Marceglia S, Mrakic-Sposta S, Foffani G (2006). Gender-related differences in the human subthalamic area: a local field potential study. Eur J Neurosci.

[63] Eisinger RS, Cagle JN, Opri E (2020). Parkinsonian beta dynamics during rest and movement in the dorsal pallidum and subthalamic nucleus. J Neurosci.

[64] Telkes I, Sabourin S, Durphy J (2020). Functional use of directional local field potentials in the subthalamic nucleus deep brain stimulation. Front Hum Neurosci.

[65] Henderson L, Kennard C, Crawford TJ (1991). Scales for rating motor impairment in Parkinson’s disease: Studies of reliability and convergent validity. J Neurol Neurosurg Psychiatry.

[66] Rupprechter S, Morinan G, Peng Y (2021). A clinically interpretable computer-vision based method for quantifying gait in Parkinson’s disease. Sensors.

[67] Stam MJ, van Wijk BCM, Sharma P (2022). A comparison of methods to suppress electrocardiographic artifacts in local field potential recordings. bioRxiv.

[68] Wang DD, de Hemptinne C, Miocinovic S (2016). Subthalamic local field potentials in Parkinson’s disease and isolated dystonia: an evaluation of potential biomarkers. Neurobiol Dis.

[69] Restrepo-Vera J, Coscojuela P, Fonseca E (2022). Epileptic seizures in the emergency room: clinical and electroencephalographic findings associated with brain perfusion patterns on computed tomography. J Neurol.

[70] Chen CC, Litvak V, Gilbertson T (2007). Excessive synchronization of basal ganglia neurons at 20 Hz slows movement in Parkinson’s disease. Exp Neurol.

[71] Chen CC, Lin WY, Chan HL (2011). Stimulation of the subthalamic region at 20Hz slows the development of grip force in Parkinson’s disease. Exp Neurol.

[72] Eusebio A, Chen CC, Lu CS (2008). Effects of low-frequency stimulation of the subthalamic nucleus on movement in Parkinson’s disease. Exp Neurol.

[73] Leblois A, Meissner W, Bioulac B (2007). Late emergence of synchronized oscillatory activity in the pallidum during progressive parkinsonism. Eur J Neurosci.

[74] Brazhnik E, Novikov N, McCoy AJ (2021). Early decreases in cortical mid-gamma peaks coincide with the onset of motor deficits and precede exaggerated beta build-up in rat models for Parkinson’s disease. Neurobiol Dis.

[75] Buijink AWG, Piña-Fuentes DA, Stam MJ (2022). Thalamic local field potentials recorded using the deep brain stimulation pulse generator. Clin Neurophysiol Pract.

[76] Swann NC, De Hemptinne C, Thompson MC (2018). Adaptive deep brain stimulation for Parkinson’s disease using motor cortex sensing. J Neural Eng.

[77] Shah A, Nguyen TAK, Peterman K (2022). Combining multimodal biomarkers to guide deep brain stimulation programming in Parkinson disease. Neuromodulation.

[78] Merk T, Peterson V, Lipski WJ (2022). Electrocorticography is superior to subthalamic local field potentials for movement decoding in Parkinson’s disease. Elife.

[79] Rosin B, Slovik M, Mitelman R (2011). Closed-loop deep brain stimulation is superior in ameliorating parkinsonism. Neuron.

[80] Johnson V, Wilt R, Gilron R (2021). Embedded adaptive deep brain stimulation for cervical dystonia controlled by motor cortex theta oscillations. Exp Neurol.

[81] Cagnan H, Pedrosa D, Little S (2017). Stimulating at the right time: phase-specific deep brain stimulation. Brain.

[82] Cernera S, Alcantara JD, Opri E (2021). Wearable sensor-driven responsive deep brain stimulation for essential tremor. Brain Stimul.

[83] Little S, Pogosyan A, Neal S (2013). Adaptive deep brain stimulation in advanced Parkinson disease. Ann Neurol.

[84] Little S, Beudel M, Zrinzo L (2016). Bilateral adaptive deep brain stimulation is effective in Parkinson’s disease. J Neurol Neurosurg Psychiatry.

[85] Petrucci MN, Neuville RS, Afzal MF (2020). Neural closed-loop deep brain stimulation for freezing of gait. Brain Stimul.

[86] Rosa M, Arlotti M, Marceglia S (2017). Adaptive deep brain stimulation controls levodopa-induced side effects in Parkinsonian patients. Mov Disord.

[87] Arlotti M, Marceglia S, Foffani G (2018). Eight-hours adaptive deep brain stimulation in patients with Parkinson disease. Neurology.

[88] Piña-Fuentes D, van Dijk JMC, van Zijl JC (2020). Acute effects of adaptive Deep Brain Stimulation in Parkinson’s disease. Brain Stimul.

[89] Gilron R, Little S, Perrone R (2021). Long-term wireless streaming of neural recordings for circuit discovery and adaptive stimulation in individuals with Parkinson’s disease. Nat Biotechnol.

[90] Swinnen BEKS, Buijink AW, Piña-Fuentes D (2022). Diving into the subcortex: The potential of chronic subcortical sensing for unravelling basal ganglia function and optimization of deep brain stimulation. Neuroimage.

[91] Hirschmann J, Schoffelen JM, Schnitzler A, van Gerven MAJ (2017). Parkinsonian rest tremor can be detected accurately based on neuronal oscillations recorded from the subthalamic nucleus. Clin Neurophysiol.

[92] Yao L, Brown P, Shoaran M (2020). Improved detection of Parkinsonian resting tremor with feature engineering and Kalman filtering. Clin Neurophysiol.

[93] Khawaldeh S, Tinkhauser G, Torrecillos F (2022). Balance between competing spectral states in subthalamic nucleus is linked to motor impairment in Parkinson’s disease. Brain.

[94] Khawaldeh S, Tinkhauser G, Shah SA (2020). Subthalamic nucleus activity dynamics and limb movement prediction in Parkinson’s disease. Brain.

[95] Neumann WJ, Memarian Sorkhabi M, Benjaber M (2021). The sensitivity of ECG contamination to surgical implantation site in brain computer interfaces. Brain Stimul.

[96] Fasano A, Gorodetsky C, Paul D (2022). Local field potential-based programming: a proof-of-concept pilot study. Neuromodulation.

